# Ability of rumen protozoa *Diploplastron affine* to utilize β-glucans

**DOI:** 10.1007/s12223-012-0120-y

**Published:** 2012-04-13

**Authors:** Grzegorz Belzecki, Renata Miltko, Tadeusz Michalowski

**Affiliations:** The Kielanowski Institute of Animal Physiology and Nutrition Polish Academy of Sciences, 05-110 Jabłonna, Poland

## Abstract

The ability of the rumen ciliates to utilize β-glucans other than cellulose and xylan is currently being recognized. The objective of the present study was to characterize the ability of the ciliate *Diploplastron affine* to digest some pachyman, laminarin, pustulan, curdlan and lichean. The protozoa were isolated from the rumen of sheep and either grown in vitro or inoculated into the rumen of ciliate-free sheep and maintained in natural conditions. In vitro culture studies showed that the enrichment of culture medium with the examined saccharides results in an increase in the number of ciliates in comparison to the control cultures. The increase was over 36 and 15 % when the growth medium was supplemented with pachyman (1,3-β-glucan) and pustulan (1,6-β-glucan), respectively. A positive correlation was also found between the population density of ciliates and the dose of saccharide supplemented to the growth medium. Enzyme studies were performed using the crude enzyme preparation obtained from ciliates treated with antibiotics. The ability of ciliates to digest the examined β-glucans was tested by the quantification of reducing sugars released from the mentioned substrates during the incubation with crude enzyme preparation. The results showed that *D. affine* ciliates were able to digest both of them. The mean degradation rate varied between 6.7 and 28.2 μmol/L glucose per mg protein per h for pustulan and lichean, respectively, whereas the digestion velocity was the highest at 5.0–5.5 pH and 45–50°C.

## Introduction

β-Glucans other than cellulose are widespread in nature. They consist of two main classes. The 1,3-β-glucans are present in numerous plants (Burton and Fincher 2009; Fincher and Stone 1986), while 1,6-β-glucans occur in fungi (Bartnicki-Garcia 1970; Beran et al. 1972; Seichertová et al. 1973) or lichens (Carbonero et al. 2006). However, the most numerous β-glucans possess a mixed type of linkages, i.e. 1,3–1,6-β- or 1,3–1,4-β-glycosidic (Martin et al. 2007).

Rumen ciliate protozoa engulf and digest small particles of cereal grains and fungal zoospores. They are also able to break down lichens and the rumen fungal mycelium. Each of the mentioned nutrients contains β-glucans. Moreover, lichen is an important component of reindeer diet (Nieminen and Heiskari 1989). However, little is known about the ability of rumen protozoa to cover their saccharide requirements by the mentioned polysaccharides. The objective of the present study was to determine the ability of rumen ciliate *Diploplastron affine* to digest the insoluble 1,3-β-glucans—pachyman—or 1,6-β-glucans such as pustulan.

## Material and methods

### Protozoa

The ciliates were identified according to Dogiel (1927). They were isolated from the natural rumen fauna of sheep and cultured in vitro or in vivo. The in vitro cultures were initiated by the picking of the individuals exhibiting morphologically typical features of *D. affine*. The picked ciliates were put into 50-mL Erlenmeyer flasks filled with 40 mL culture of rumen bacteria growing on “*caudatum*” salt solution (Coleman et al. 1972). The solution consisted of (in grams per litre): K_2_HPO_4_ 6.3, KH_2_PO_4_ 5.0, CaCl_2_·6H_2_O 0.09, MgSO_4_·7H_2_O 0.09, CH_3_COONa 0.75, NaCl 0.65 and distilled water. About 50 cells of protozoa were put into each vessel. The ciliates were cultivated by the method routinely used in the laboratory (Michalowski et al. 1986). If necessary, they were transferred to a continuous culture system (Michalowski 1979), and after population development, they were inoculated into the rumen of ciliate-free sheep and cultured in vivo to obtain material for enzyme studies. The elimination of the natural ciliate fauna from the rumen of sheep was made by the washing procedure described by Michalowski et al. (1999).

### Cultivation experiment

To determine the influence of 1,3-β-glucan on the population density of *D. affine*, the protozoa were grown in vitro and their number was determined. The culture medium of control cultures was composed of “*caudatum*” salt solution, powdered meadow hay and wheat gluten. The growth medium of the experimental cultures consisted of the same components supplemented with pachyman (1,3-β-glucan) or pustulan (1,6-β-glucan). Meadow hay and wheat gluten were supplied in the proportion of 0.3 and 0.08 mg/mL culture per day, respectively. The examined β-glucans were added in four different doses, i.e. 0.015, 0.030, 0.045 and 0.060 mg/mL culture per day. The cultures of ciliates were fed every day and were transferred every fourth day on the fresh medium according to Michalowski et al. (1986). Three cultures were run simultaneously on each kind of the culture medium. The cultivation period lasted 28 days, and the ciliates were counted microscopically for each dilution and on each day according to Michalowski et al. (1986). Briefly, the material that remained after the transfer was thoroughly mixed, sampled and preserved by an addition of an equal volume of 4 % aqueous solution of formaldehyde. The counting of ciliates was done by the method routinely used in the laboratory. A 0.1-mL sample of the preserved material was poured on a microscopic slide, and all morphologically intact ciliates were counted. Three samples of the same material were always counted, and the mean value was calculated.

### Enzyme studies

The protozoa growing in the rumen of sheep (see above) were used to perform the enzyme studies. Samples of rumen content (mass of about 1 kg) were withdrawn from the rumen. They were diluted with 2 L of “*caudatum*” salt solution (see above) and squeezed through a screen of 260 μm pore size. The filtrate was collected, poured into a separating funnel and allowed to stand at 40°C for about 30 min. The protozoa forming a white pellet at the bottom of the funnel were collected and purified with the use of a sedimentation method. The crude enzyme preparation was prepared from ciliates following their incubation with antibiotics. This was done in order to eliminate the intracellular bacteria. Due to this the purified protozoa were suspended in a warm (40°C) salt solution of “*caudatum*”. The suspension was supplemented with chloramphenicol, streptomycin and ampicillin and incubated for 18 h at 40°C. The final concentration of particular antibiotics in the suspension of ciliates was 50 μg/mL. After incubation, the ciliates were washed as described above and immediately used to prepare the crude enzyme preparation, or were stored at −80°C. This enzyme preparation was obtained by the homogenization of ciliates and centrifugation of the homogenate at 22,000×*g* to remove unbroken ciliate cells and their fragments. The ability of ciliates to degrade the examined β-glucans was determined by the quantification of reducing sugars released following their incubation for 1 h with the crude enzyme preparation (Miller et al. 1960). The reaction mixture consisted of 0.4 mL 0.2 % solution of substrate, 0.4 mL crude enzyme preparation and 0.2 mL of 0.1 mol/L citrate–phosphate buffer. The reducing sugars were measured at 560 nm using a Beckman DU 64 spectrophotometer.

### Statistical analysis

Student’s *t* test was used to compare the differences between mean values according to Ruszczyc (1970).

## Results and discussion

### The effect of 1,3-β- and 1,6-β-glucans on the population density of *D. affine*

The in vitro studies were done to examine the role of β-glucans in development of the population of *D. affine*. To perform this experiment, pachyman and pustulan were selected because they are composed of β-d-glucose, the residues of which are completely bound by 1,3-β- or 1,6-β-glucosidic linkages, respectively (Bartnicki-Garcia 1970; Burton and Fincher 2009; Carbonero et al. 2006; Fincher and Stone 1986). The concentration of *D. affine* cells cultured on medium supplemented with pachyman or pustulan varied from 1,000 to 3,110 and from 1,722 to 3,110 ciliates per millilitre, in relation to the day and diet, respectively. The mean data obtained during 28 days of cultivation of ciliates are presented in Table 1. They showed that an increase in the dose of supplemental saccharides was accompanied by an increase in the ciliate number; a positive correlation between the two compared parameters was confirmed by the statistical calculations in the case of pachyman (*r* = 0.567; degrees of freedom 0.587; *p* < 0.01) and pustulan (*r* = 0.589; degrees of freedom 0.589; *p* < 0.01). The results showed that supplementation of growth medium with both β-glucans resulted in statistically significant increase in the numbers of ciliates in comparison to the control cultures (*p* < 0.05). This increase varied between 22 and 42 % and 10 and 18 % in the case of pachyman and pustulan, respectively. No significant effect was stated only when the lowest dose of the latter saccharide was applied. Thus in general, the response of *D. affine* ciliates to the supplementation of culture medium with β-1,3- and β-1,6-glucans was similar with that observed earlier in the case of crystalline cellulose (Michalowski et al. 1986) or murein (Belzecki et al. 2010). Thus the findings suggest that insoluble glucan improved the growth conditions required by ciliates. One of the possible explanations of this finding seems to be an enrichment of culture medium with constituents which are digested and utilized by the examined protozoa as a source of energy.

**Table 1:** The mean concentration of *D. affine* ciliates cultivated in vitro in a medium supplemented with different doses of pachyman and pustulan

Type of glucan	Diet^a^				
	A	B	C	D	E
Pachyman	1,860a	1,952b	2,273b	2,425b	2,525b
Pustulan	1,999a	2,109a	2,186b	2,280b	2,335b

Values with a different letter differ significantly at *p* < 0.01

^a^A—control diet: hay (0.3 mg/mL per day) and wheat gluten (0.08 mg/mL per day); B, C, D and E—experimental diet consisting of control diet supplemented with 0.03, 0.06, 0.12 and 0.24 mg/mL per day of glucan, respectively

### Enzymic studies

Apart from pustulan and pachyman, three other β-glucans were used to study the digestion ability of the ciliates. The incubation of crude enzyme preparation of rumen ciliate *D. affine* with five different types of β-glucans resulted in the release of reducing sugars from each of them (Table 2). However, the degradation rate differed significantly in relation to the digested substrate. Of the tested saccharides, lichean was digested with the highest velocity while pustulan with the lowest. The relevant values were 28.2 and 6.7 μmol/L released glucose per mg protein per h, respectively. The comparison of the remaining data showed that the degradation rate of pachyman, curdlan, laminarin and pustulan was about 70, 50, 35, and 25 % lower than that of lichean, respectively. The optimum conditions to digest the examined saccharides varied between 5.0 and 5.5 pH and 45 and 50°C. There are only few data concerning of the ability of other species of rumen ciliates to degrade β-glucans other than cellulose or xylan. For example, Bailey and Gaillard (1965) examined the ability of *Epidinium ecaudatum* to digest laminarin. Similar investigations were performed by Morgavi et al. (1994) on the mixed rumen fauna of goats. A comparison of their results with the results reported here showed that the laminarin-degrading enzymes of mixed protozoa population released almost two times less glucose from this substrate than the crude enzyme preparation of *D. affine*. It is also noteworthy to emphasize that the ability of rumen ciliates to digest the other β-glucans has not yet been described. Thus our results characterize such ability for the first time.

**Table 2:** The optimum conditions and digestion rate [mean values (*n* = 3)] of different β-glucans by crude enzyme preparation of *D. affine*

Saccharide	pH	Temperature (°C)	Degradation rate (μmol/L glucose per mg protein per h)
Pachyman (1,3-β-glucan)	5	45	20.2a
Laminarin (1,3–1,6-β-glucan)	5.5	50	10.2b
Pustulan (1,6-β-glucan)	5	55	6.7c
Curdlan (1,3-β-glucan)	5	55	15.3d
Lichean (1,3–1,4-β-glucan)	5.5	50	28.2e

Values with different letter differ significantly at *p* < 0.01

Because of the complex structure of β-glucans (Martin et al. 2007), the enzymes responsible for their degradation belong to four classes of enzymes. The first of them is 1,3–1,4-β-d-glucan 4-glucanohydrolase (EC 3.2.1.73) which catalyses the 1,4-β-glucosidic linkages and is commonly termed lichenase or licheninase. The second class is 1,6-β-d-glucan glucanohydrolase (EC 3.2.1.75) randomly hydrolysing the 1,6-β-glucosidic linkages and named pustulanase. Finally, the third class is endo-1,3(4)-β-glucanase (EC 3.2.1.6). It hydrolyzes the 1,3- or 1,4-β-glucosidic linkages inside of the chain of glucose residues and is commonly termed laminarinase. The last class contains the endo-1,3-β-glucanase (EC 3.2.1.39) hydrolyzing the 1,3-β-glucosidic linkages. Unfortunately, the studies presented in this report are of preliminary character and do not include the identification and characterization of enzymes involved in the digestion of particular β-glucans listed in such studies. This identification will be undertaken by our group in the near future.
